# Supplementary material for: TALEN-Mediated Gene Targeting for Cystic Fibrosis-Gene Therapy
Source: Genes (Basel). 2019 Jan 11;10(1):39. doi: 10.3390/genes10010039 (PMC6356284; doi:10.3390/genes10010039)
Supplement: Supplementary file 1 [file genes-10-00039-s001.pdf]

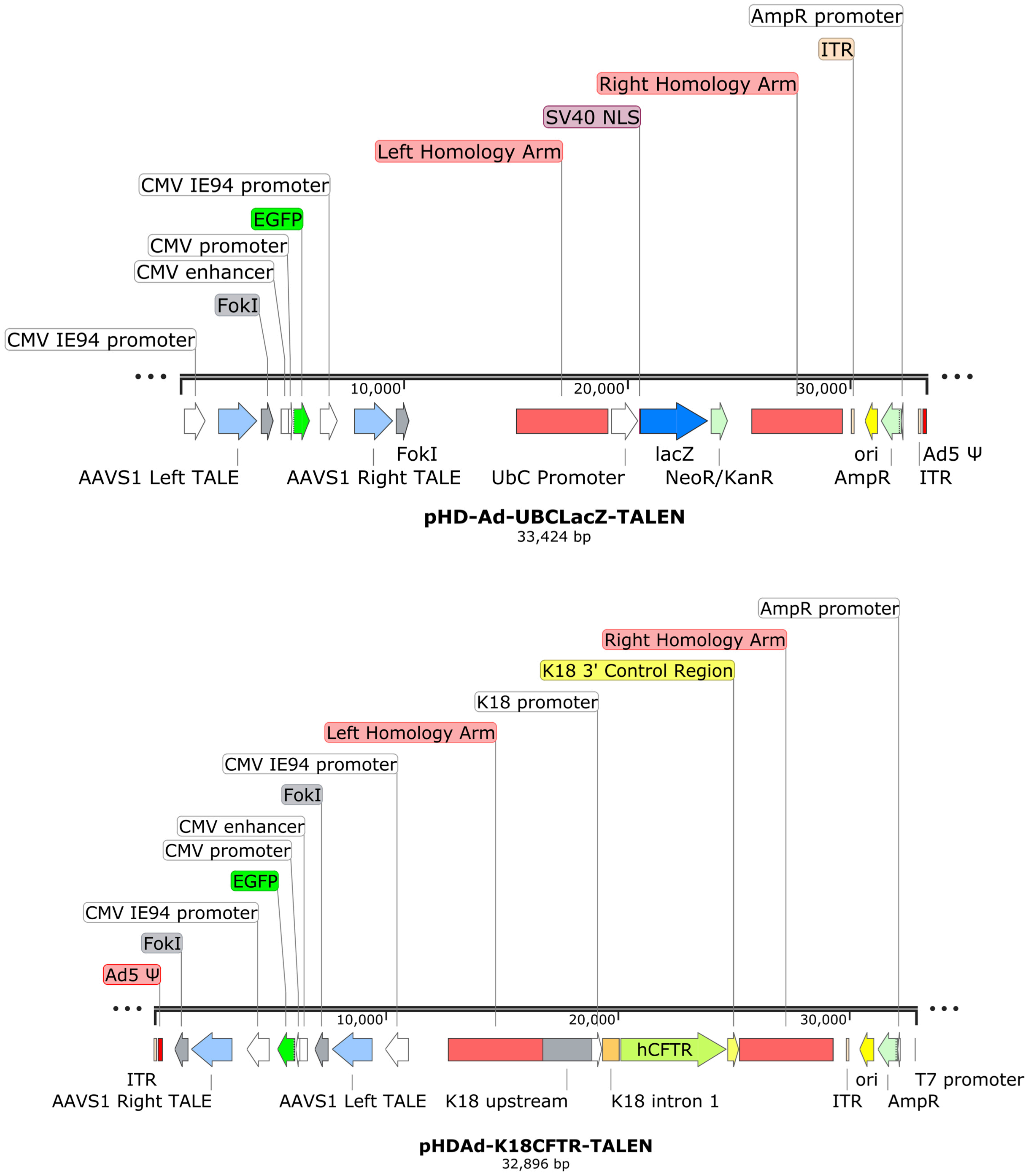

Supplementary Figure 1. Vector maps for HD-Ad-K18CFTR-TALEN and HD-Ad-UBCLacZ- TALEN. Two TALEN homodimers driven by CMV IE94 promoter were labeled in blue; CMV-EGFP genes (labeled in green) were cloned in between two TALEN dimers. LacZ-neo fusion gene (labeled in blue) was driven by UBC promoter and was flanked by 4 Kb homology arms (in red). The hCFTR gene was driven by K18 promoter and intron and was flanked by 4 kb homology arms (in red). The ITR and Ad5 packaging signal were part of HD-Ad vector backbone.

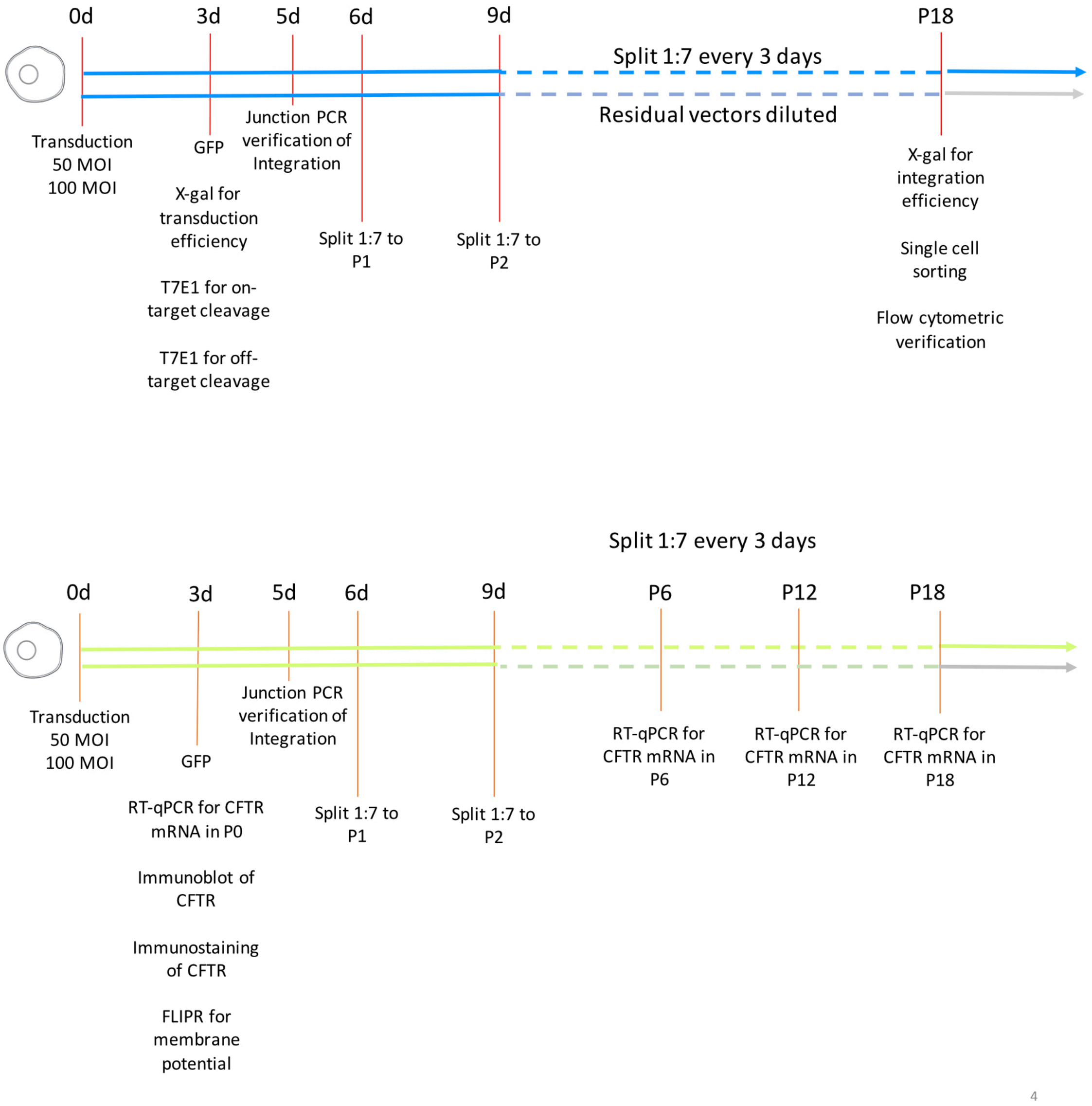

Supplementary Figure 2. Schematic diagram of experimental designs and time courses for data collection.

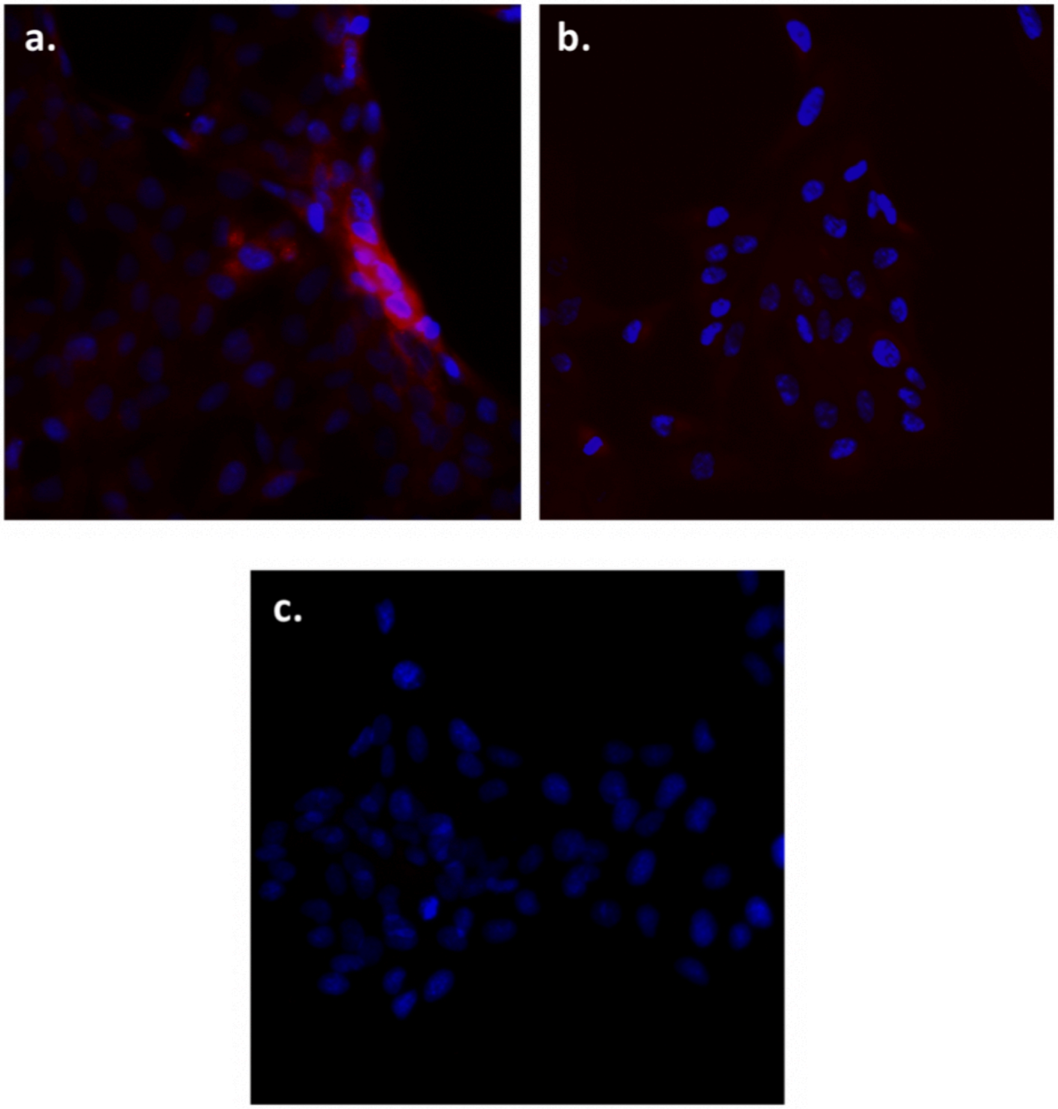

Supplementary Figure 3. Immunodetection of human CFTR expression. (A). Immunofluorescent staining for CFTR (red) in IB3-1 cells transduced with 100MOI of HD-Ad-K18CFTR vector and cultured for 3 days. (B). Immunofluorescent staining for un-transduced IB3-1 cells. (C). No antibody control. Cell nuclei were labeled using DAPI.

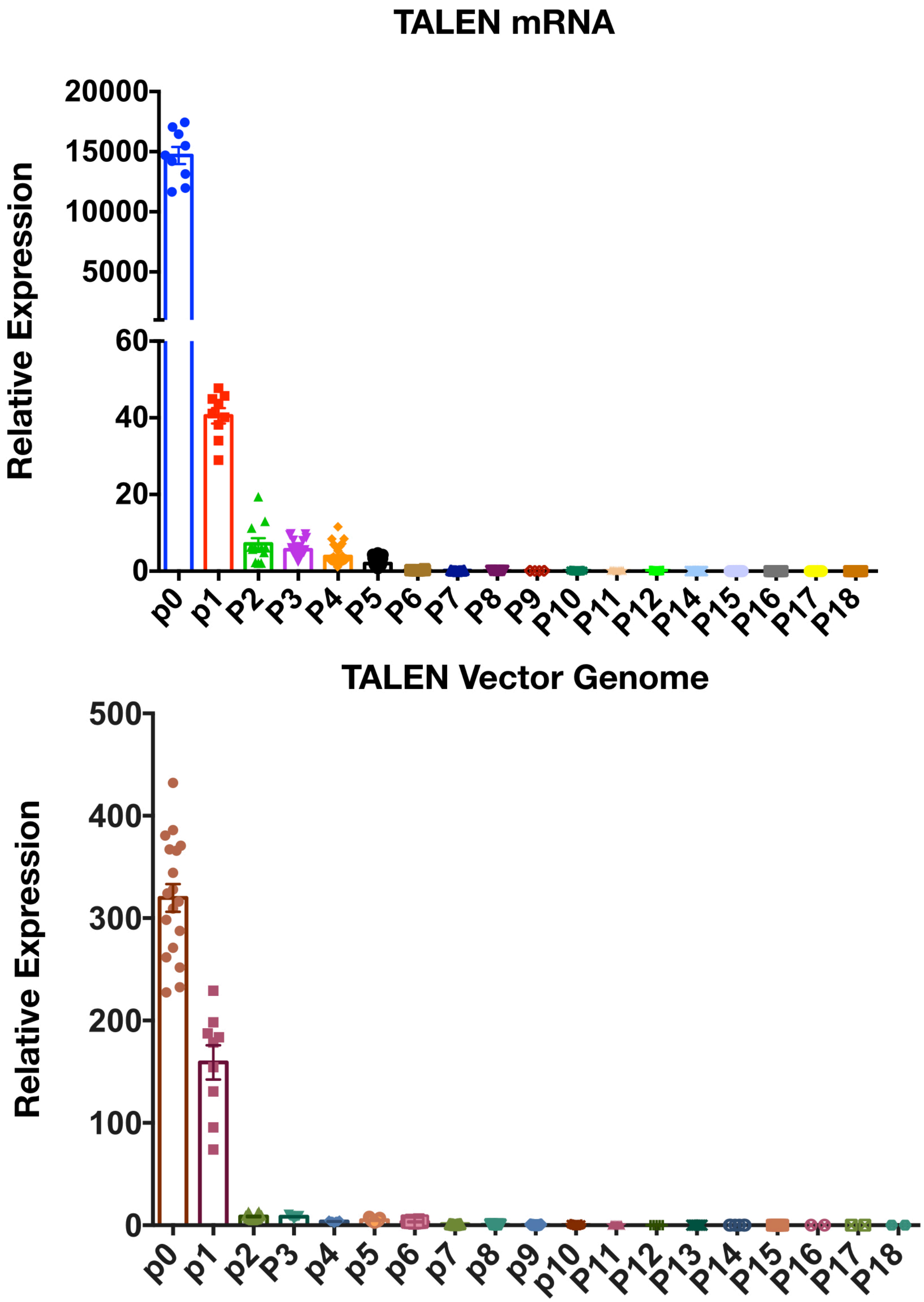

Supplementary Figure 4. Top, qPCR analysis for TALEN mRNA level at each passage for 18 passages.IB3-1 cells transduced with 100MOI of HD-Ad-K18CFTR-TALEN were passaged at an 1:7 ratio for 10 generations; for each passage, total RNA was extracted from 1 x 10^6 cells for qPCR analysis. Primers used for TALEN mRNA detection are 5'GAGAACCAGACCCGGAATAAG (forward primer), 5' GCCTTGTAGTTGCCCTTGA (reverse primer). Bottom, relative quantity of TALEN vector genome level measured using qPCR. Cellular genome from IB3-1 cells transduced with 100MOI of HD-Ad-K18CFTR-TALEN vector at each passage for 18 passages. qPCR analysis was performed using primers against HD-Ad packaging signal.
